# Drivers of improved prognosis in pediatric sepsis in China: policy support and collaborative actions

**DOI:** 10.3389/fped.2026.1874411

**Published:** 2026-07-23

**Authors:** Gang Liu, Boliang Fang, Suyun Qian

**Affiliations:** Department of Pediatric Intensive Care Unit, Beijing Children's Hospital, Capital Medical University, National Center for Children's Health, Beijing, China

**Keywords:** child, collaborative action, low resource, policy support, sepsis

## Abstract

Sepsis represents a critical threat to child health, with diagnostic and treatment capabilities remaining especially constrained in resource-limited settings. In response to disparities in healthcare development, China has, over the past decade, implemented an integrated model of policy support and collaborative action, leading to measurable improvements in pediatric sepsis management and a notable decline in associated mortality. Policy initiatives have laid the essential groundwork for systemic capacity building, guiding sustained investment in healthcare infrastructure and facilitating the equitable distribution of medical expertise. Concurrently, coordinated professional actions—including guideline dissemination, establishment of pediatric care networks, and targeted training programs—have standardized clinical practice and strengthened frontline response. Drawing on field insights and available evidence, this article outlines Chinàs dual-track approach at the policy and operational levels, offering a national perspective on sepsis control that may inform global efforts.

## 1 Introduction

Globally, healthcare systems in resource-limited settings face significant structural challenges, with pediatric sepsis care often constrained by inadequate diagnostic facilities, insufficient organ support capacity, and delays in updating clinical knowledge and skills among medical teams (1, 2). Supporting and improving the diagnosis and management of pediatric sepsis in resource-limited settings remains a priority for international pediatric critical care organizations (3, 4). In the early 2000s, the first Chinese multicenter study on pediatric sepsis reported an in-hospital mortality rate of 34.6% among children with severe sepsis (including septic shock) (2010–2011) (5). During a comparable period (2008–2009), two better-equipped tertiary children's hospitals in Beijing recorded a in-hospital mortality rate of 28.7% (6), while the U.S. national mortality rate for pediatric severe sepsis as early as 1995 was only 10.3%, highlighting a substantial gap (7).

After more than a decade of sustained efforts, China has achieved marked improvements. Recent data indicate that in-hospital mortality from pediatric severe sepsis (including septic shock) was 16.7%–18.8%, among children with septic shock has dropped from 28.9% to 18.3%–23.4% (6, 8–10). A multicenter study conducted in the southwestern region of China, involving provincial and selected municipal hospitals, reported a 19.2% fatality rate among pediatric patients with septic shock (10). We attribute the improving trends in sepsis outcomes primarily to policy support and industry initiatives aimed at enhancing medical conditions. This article outlines China's initiatives and outcomes in this regard, offering practical insights to inform global strategies against pediatric sepsis.

## 2 Policy-driven infrastructure development: laying the foundation for pediatric sepsis care in resource-limited settings

Over the past two decades, the Chinese government has issued a series of policy documents, including the *National Program for Child Development (2001–2010)*, *the National Program for Child Development (2011–2020)*, and *the Healthy Child Action Plan (2021–2025)*. These policies strengthen the healthcare system nationwide, especially in resource-limited areas. From 2016 to 2020, the central government allocated a total of 141.5 billion CNY to support 6,348 healthcare infrastructure projects across the country, including 2,085 county-level hospitals, which received 72.2 billion CNY (49.8%) (11). In the 15th national top-level development plan released in 2026, the government reiterates the commitment to enhancing medical capacity at the primary level and in low-resource areas. Consistent and strong policy support has driven sustained investment, fostered healthcare system development, and the institutionalized downward deployment of high-quality medical resources. On the historical page of the WFPICCS, Chinese colleagues noted that in 2014, there were over 200 pediatric intensive care units (PICUs) in China (12). In 2024, China issued *the Opinions on Strengthening Critical Care Medicine Service Capacity*, launching a government-led, nationwide initiative to enhance pediatric critical care capacity.

Within a year of its implementation, the number of PICUs has increased substantially. Moreover, a “paired assistance” model has been adopted, where top-tier hospitals provide government-led targeted support to some hospitals in resource-limited areas to improve their clinical care. For example, in the remote Yushu Tibetan Autonomous Prefecture in Qinghai Province, the largest local general hospital has received long-term, on-site assistance from top-tier hospitals in Beijing, including the National Children's Medical Center (Beijing). This support includes the long-term dispatch of medical teams and the ongoing upgrade of a 15-bed ICU capable of serving children of all ages. Beijing Children's Hospital has successively provided paired assistance to 23 hospitals. Among them, four primary care facilities are located in remote areas such as Inner Mongolia and Qinghai, and four are provincial hospitals that are intended to become national regional medical centers. At present, approximately 300 medical and managerial staff are dispatched on average every month. According to reports from the National Health Commission of the People's Republic of China, a total of 1,173 tertiary hospitals across China have been organized to deliver paired support to 1,496 county-level hospitals covering 940 counties. Over 180,000 person-times of medical personnel have been deployed, providing 48 million person-times of medical services to the public. Many primary pediatric institutions across China are directly benefiting from such collaborative initiatives. Although specific data are still scarce, many pediatric practitioners involved in these partnerships report tangible improvements in diagnostic and treatment capabilities at these medical institutions.

## 3 Proactive actions in the medical community drive improvements in pediatric sepsis care

The pediatric critical care community has actively promoted the localized implementation of international sepsis guidelines. Chinese consensuses documents were promptly issued in 2006, 2015, and 2025, closely following corresponding international guidelines updates (13–15). This rapid response enabled timely integration of evidence-based practices into routine clinical care. A retrospective study comparing guideline adherence across three pediatric hospitals of different tiers reported no significant difference in 6-h resuscitation target achievement or the proportion of patients receiving antibiotics within the first hour after diagnosis, suggesting that standardized sepsis management could be effectively promoted across varying resource settings (16). Furthermore, the pediatric sector in China, has independently established multi-level medical alliances that link higher-level institutions with primary care facilities. These networks facilitate the dissemination of updated knowledge and skills to clinicians in underserved areas. In addition, large national and provincial medical centers organize extensive, tiered training programs focused on pediatric infection and sepsis each year, with an emphasis on improving early recognition and management. They also train healthcare workers from resource-limited regions through medical advanced study programs. Taking the PICU of National Center for Children's Health (Beijing) as an example, it annually accepts medical staff from ethnic minority areas, remote regions, and primary facilities for training in sepsis, basic skills, and the use of organ support equipment.

## 4 Discussion

Critical care management of critically ill patients in globally resource-limited regions is frequently confronted with multiple challenges, including inadequate infrastructure, shortages of disposable medical supplies, and insufficient quantity or professional competency of healthcare workers. Scaling up government investment to support the development of the primary healthcare service system serves as an effective coping strategy (17). A Nigerian study on reducing in-hospital cardiopulmonary arrest identified that shortages of ICU beds, inefficient access protocols for emergency medications, and inadequate incentives for medical staff were major barriers to the implementation of in-hospital rapid response systems (18). Intensive care resources are severely scarce and unevenly distributed in low- and middle-income countries (LMICs), resulting in a substantial number of patient deaths during inter-hospital transportation (19). Stephen A. Spencer and colleagues proposed that low-income countries should prioritize ensuring equitable access to essential critical care services, while addressing systemic deficiencies in workforce capacity, clinical data infrastructure, pharmaceuticals and medical devices, healthcare financing, and governance (20). Over the past two decades, driven by strong policy and financial support, China has witnessed accelerated development of professional teams and medical devices for critical care, including pediatric intensive care units (PICUs). Meanwhile, standardized criteria for ICU construction and operational optimization have been formulated under industry consensus, which constitutes the fundamental driver for the improvement of pediatric sepsis care quality (21, 22).

China's healthcare industry has long implemented a hospital-to-hospital targeted paired assistance policy, and the effectiveness of this vertical support model is well evidenced by international precedents. Rwanda leveraged the Population Health Implementation and Training (PHIT) international cooperation program to address systemic deficiencies including weak primary healthcare delivery, inadequate medical quality management, and imperfect assessment mechanisms for health workforce professionals. Under government leadership, Rwanda launched a two-year structured support initiative, which reconstructed its local health system and established a replicable model for rural healthcare upgrading (23). Australia and Indonesia have built a long-term, institutionalized bilateral cooperation framework, which has substantially improved the quality of clinical nursing and emergency care in Indonesian partner hospitals (24). Although direct clinical evidence confirming the impact of such assistance on improving the prognosis of pediatric sepsis remains limited, we argue that this resident, long-term vertical support model is more effective in substantively elevating primary care facilities diagnosis and treatment capacity than academic exchanges or short-term *ad hoc* training programs alone. Mortality rate of children under-five in China fell from 10.2 per thousand to 6.8 per thousand between 2016 and 2022 (25). In the context of sepsis, improving the standardization of bundle care may be a priority for enhancing the quality of diagnosis and treatment.

## 5 Conclusion

In summary, notwithstanding China′s uneven development of healthcare resources, sustained policy support and proactive advances in the medical field have substantially strengthened nationwide pediatric critical care capacity. Such systematic improvement serves as a core contributor to the improved clinical prognosis of children with sepsis across China. In the future, core interventions to enhance sepsis care in resource-limited regions consist of strengthening local clinician training, expanding telemedicine outreach from tertiary hospitals, and gradually integrating primary medical institutions into the national disease quality control framework, et al. Nevertheless, it should be noted that the findings presented in this study are primarily derived from domestic real-world practice and regional clinical experiences, with direct evidence supporting definitive causality still limited. Despite this limitation, the experience and insights summarized herein may provide practical references and actionable implications for global peers engaged in the prevention and management of pediatric sepsis.

## Data Availability

The original contributions presented in the study are included in the article/Supplementary Material, further inquiries can be directed to the corresponding author.
